# Biochemical and transcriptomic profiling analysis of drought tolerant related genes in ML 82-2 and ML 125-2 rice mutant lines

**DOI:** 10.5114/bta/200704

**Published:** 2025-03-31

**Authors:** Jun Ying Low, Rhun Yian Koh, Hussein Sobri, Ahmad Faiz, Anna Pick Kiong Ling

**Affiliations:** 1 Division of Applied Biomedical Sciences and Biotechnology, School of Health Sciences, IMU University, Kuala Lumpur, Malaysia; 2 Agrotechnology and Biosciences Division, Malaysian Nuclear Agency, Selangor, Malaysia

**Keywords:** biochemical analysis, drought tolerant rice, gene ontology enrichment analysis, mutation breeding, transcriptomic profiling analysis

## Abstract

**Background:**

Backcrossing of gamma-irradiated IS21 with MR220 has generated progenies (ML 82-2 and ML 125-2) with improved traits. However, studies on these new mutant lines remain limited. This study aimed to determine and compare the biochemical characteristics and transcriptomic profiles of drought-tolerance-related genes in ML 82-2 and ML 125-2, as well as in the parent lines, IS21 and MR220.

**Materials and methods:**

Seeds were germinated for 14 days under a controlled photoperiod (16 h light and 8 h darkness) at a constant temperature of 25 ± 2°C. Biochemical analyses, including total soluble protein content, specific peroxidase activity (SPA), chlorophyll content, and proline content, were conducted. Transcriptomic profiling was performed using STRING and gene ontology (GO) enrichment analysis.

**Results:**

ML 82-2 exhibited the highest SPA, which was significantly different from that of MR220 and IS21, as well as significantly different total soluble protein content. However, ML 82-2 did not significantly differ from MR220 in chlorophyll and proline content. Drought stress-responsive genes *Os01g0124401, Os08g0473900,* and *Os08g0518800* were identified in ML 82-2. Conversely, ML 125-2 displayed total soluble protein content and SPA similar to IS21, while chlorophyll and proline content were not significantly different from those of MR220. Drought stress-responsive genes *Os10g0471100, Os01g0197100,* and *Os11g0701400* were identified in ML 125-2.

**Conclusions:**

ML 82-2 demonstrated improved SPA, whereas ML 125-2 exhibited enhanced total soluble protein content. The identified genes in both mutant lines are associated with drought resistance, with most sharing a similar genomic profile with MR220. These findings contribute to plant physiology studies and stress-responsive gene discovery in rice.

## Introduction

Rice (*Oryza sativa*) is the staple food for nearly half of the global population (FAO, 2023). In 2023/2024, world rice production was forecasted to reach 523.9 million tonnes, according to the Food and Agriculture Organization of the United Nations (FAO, 2023). To meet the growing global population and demand, *O. sativa* production is expected to increase by at least 25% by 2030 (Rahman et al., 2023). However, statistics indicate that 820 million people worldwide still suffer from hunger (Rigillo et al., 2021), while rice remains a staple food for more than 3.5 billion people (Dorairaj et al., 2023).

With a 40% share in the international rice market, India is the world’s largest rice exporter. However, India’s ban on nonbasmati white rice exports on July 20, 2023 – coinciding with Russia’s withdrawal from the Black Sea Grain Initiative – exacerbated shortages in global rice exports and inventories, further impacting developing countries (Valera, 2024; Sharma, 2023). As of August 2023, FAO reported a 31% surge in global rice prices compared to previous years, marking the highest increase in nearly 15 years (Abuza, 2023). Studies have consistently shown that Malaysia’s rice production meets only 65% to 75% of its self-sufficiency level (SSL) (Rahim et al., 2017). While Malaysians consume 2.7 million tonnes of rice annually, 30% of the country’s supply was imported from neighboring countries as of 2016 (Mahavera, 2023).

Beyond population-driven demand, *O. sativa* faces multiple abiotic stress factors, including drought, salinity, extreme temperature fluctuations, and UV radiation, along with biotic stress factors such as pests and diseases (Hussain et al., 2020; Raza et al., 2019). Recently, temperature fluctuations and adverse weather conditions, particularly El Niño, have significantly affected Malaysia’s paddy harvest in Tanjung Karang, Sekinchan, and Sabak Bernam, Selangor. This led to a delayed harvest, shifting from the scheduled July– August period to October, and resulted in a 20% decline in rice production (Khoo, 2023). In summary, the combined pressures of population growth and environmental factors continue to impact global rice production and food security.

Physical radiation induces a broad spectrum of mutations across a wide range of plant materials (Maghuly et al., 2016). The treated plants remain organic and free of chemical residues (Maghuly et al., 2016; Tadele, 2016). Physical radiation comprises various types, including gamma radiation, X-ray radiation, and particle radiation. The Malaysian Nuclear Agency employed gamma radiation to develop IS21, which was subsequently backcrossed with MR220 to produce new mutant lines (ML 82-2 and ML 125-2) with improved agronomic traits. Gamma irradiation was chosen as the mutation breeding method due to its high-energy photons, which penetrate plant structures and alter genes within a short period (Baadu et al., 2023; Ma et al., 2021).

Biochemical parameters are essential for measuring the enzymatic activities of plants under drought stress, as morphological and physiological assessments alone are insufficient (El-Mouhamady et al., 2022). Plants produce distinct biological and chemical metabolites to regulate biosynthetic pathways. In rice, metabolic genome-wide association studies (GWAS) have identified 840 metabolites, based on approximately 6.4 million single nucleotide polymorphisms (SNPs) from 529 rice accessions (Chen et al., 2014). Notably, enzymatic and metabolic activities in plants fluctuate depending on photosynthetic limitations, underscoring the significance of biochemical parameters in stress responses (Xiong et al., 2018). By analyzing different biochemical parameters, researchers can gain insights into plant physiology, metabolic pathways, and osmolytes involved in stress tolerance and adaptation.

Furthermore, transcriptomics profiling aims to identify genetic variations influencing metabolite expression across different rice subspecies (Chen et al., 2014). Understanding transcriptomic changes due to mutation can provide deeper insights into the molecular mechanisms underlying phenotypic changes in rice mutant lines. In this study, genomic profiling of both parent and progeny lines may reveal functional annotations and distinct gene expression patterns. The identification of drought-resistant genes in novel rice lines could facilitate downstream validation experiments and inform future targeted breeding strategies. A comprehensive understanding of differentially expressed genes (DEGs) and their role in stress-responsive biological pathways may contribute to marker-assisted backcross breeding for developing rice mutant lines with desirable quantitative trait locus (QTL) (Bashir et al., 2014).

This study aims to validate the presence of drought-stress-resistant traits in the newly developed mutant lines ML 125-2 and ML 82-2 through biochemical and transcriptomic profiling. The findings may confirm the presence of drought-tolerance-related genes and biochemical characteristics in these rice mutant lines.

## Materials and methods

### Preparation of plant materials

IS21, developed through mutation breeding via gamma radiation, was backcrossed with MR220 to produce rice mutant lines. Following preliminary screening of their physical characteristics, ML 82-2 and ML 125-2 were identified as having improved agronomic traits compared to the parent lines. Seeds for both the parent and mutant lines were provided by the Malaysian Nuclear Agency, Bangi, Selangor, Malaysia.

### Preparation and germination of seeds

The seeds were washed by inversion in a test tube filled with sterile distilled water five to six times, followed by overnight immersion in fungicide to remove surface impurities and prevent fungal growth during germination. The seeds were then planted on sterilized cotton in flasks or test tubes, with six seeds per flask and one seed per test tube. Each batch of seeds was germinated entirely in either flasks or test tubes to ensure consistency. Four replicates were prepared for each *O. sativa* line. The seeds were germinated under a controlled photoperiod of 16 h of light and 8 h of darkness at a constant temperature of 25 ± 2°C. After 14 days of growth, the seedlings were subjected to further analysis.

### Biochemical analysis of O. sativa seedlings

#### Sample extraction

Fourteen-day-old leaf samples from both parent and mutant lines were homogenized in an ice bath with the gradual addition of 1.5 ml of Tris protein extraction buffer (pH 8.0). The crude extracts were centrifuged using an Eppendorf Centrifuge 5427 R (Eppendorf, Germany) at 12,000 rpm for 20 min at 4^o^C. After centrifugation, the supernatant was collected for total soluble protein content and specific peroxidase activity analysis.

#### Determination of total soluble protein content

The Bradford method (Bradford, 1976) was used to determine total soluble protein levels. The assay was prepared by mixing 20 μl of sample extract, 80 μl of protein extraction buffer, and 5 ml of protein reagent, followed by vortexing. Absorbance was measured at 595 nm using a Shimadzu UV1800 2210V UV-Vis spectrophotometer (Shimadzu, Japan). The total soluble protein content was determined using a standard curve, with bovine serum albumin (BSA) (Sangon Biotech, China) at concentrations of 0, 2, 4, 6, 8, and 10 mg/ml as standards. Results were expressed as milligrams per gram of fresh weight (mg/g FW) of the plant material.

#### Determination of specific peroxidase activity

The method by Kokkinakis and Brooks (1979) was used to determine specific peroxidase activity. This assay is based on the oxidation of guaiacol by peroxidases in the presence of hydrogen peroxide (H_2_O_2_). The peroxidase assay was conducted by adding 500 μl of sample extract to a reaction mixture containing 7.5 ml of 0.1 M sodium phosphate buffer (pH 6.1), 1 ml of 30% H_2_O_2_ (R&M Chemicals, UK), and 1 ml of 1% guaiacol (Sangon Biotech, China). After mixing, changes in absorbance were measured at 420 nm over a 3-min interval using a Shimadzu UV1800 2210V UV-Vis spectrophotometer (Shimadzu, Japan). The initial absorbance at 0 min and the maximum absorbance recorded within the 3-min period were used to compute specific peroxidase activity, expressed in units per milligram (U/mg) of soluble protein.

#### Determination of chlorophyll content

The Lichtenthaler method (1987) was used to determine chlorophyll content. Leaf samples were ground in an ice bath at 4°C using a pestle and mortar, with 2 g of calcium carbonate (CaCO_3_) (R&M Chemicals, UK) added per gram of plant material. The mixture was centrifuged using an Eppendorf Centrifuge 5430 R (Eppendorf, Germany) at 7,000 rpm for 10 min at 4°C. After centrifugation, the supernatant was diluted with 80% acetone (R&M Chemicals, UK) to a final volume of 2.5 ml. Absorbance was measured at 646.8 and 663.2 nm using a Shimadzu UV1800 2210V UV-Vis spectrophotometer (Shimadzu, Japan). The concentrations of chlorophyll a (C_a_) and chlorophyll b (C_b_) were calculated and expressed in mg/g FW.

#### Determination of proline content

The Bates method (Bates, Waldren, and Teare, 1973) was used to determine proline content. Leaf samples were homogenized with 5 ml of 3% sulfosalicylic acid (Boer, China) and centrifuged using an Eppendorf Centrifuge 5430 R (Eppendorf, Germany) at 7,000 rpm for 10 min at room temperature. The supernatant was transferred to a new test tube and vortexed with acid ninhydrin (R&M Marketing, UK) and glacial acetic acid (R&M Chemicals, UK) in a 1 : 1 : 1 ratio. The mixture was heated at 100°C for 1 h in aluminum foil-covered tubes, followed by the addition of 1 ml of toluene (R&M Chemicals, UK). After mixing, the sample was cooled in an ice bath for 5–10 min. The absorbance of the reddish-pink upper phase was measured using a Shimadzu UV1800 2210V UV-Vis spectrophotometer (Shimadzu, Japan), with toluene as the blank. A standard curve was prepared using proline (Acros Organics, USA) at concentrations of 0, 0.002, 0.004, 0.006, and 0.010 mg/ml to calculate total soluble proline content, expressed in mg/g FW.

## Transcriptomics analysis

### RNA extraction and RNA-sequencing analysis

Total RNA was extracted using the RNeasy Plant Kit (Qiagen, Germany). Samples were homogenized and lysed in QIAshredder columns, after which the lysates were transferred onto the RNeasy silica membrane following ethanol addition. Impurities were removed through three wash steps, yielding pure and concentrated total RNA in water.

The TruSeq RNA Sample Preparation Kit (Illumina, USA) was used to construct Illumina cDNA libraries. After adapter ligation, cDNA libraries underwent DNA amplification for subsequent sequencing. The prepared cDNA libraries were then loaded onto the Illumina HiSeq2500 platform for sequencing. Library quality was assessed using Qubit and real-time PCR for quantification, while a bioanalyzer was used to determine size distribution.

Differential expression analysis was performed to identify DEGs, with statistical significance set at a false discovery rate (FDR) ≤ 0.1, *p* < 0.05, and log2 Fold Change (log_2_FC) ≥ ±2. RNA extraction and sequencing were conducted by Apical Scientific Sdn Bhd, and the raw sequencing data were deposited in the National Center for Biotechnology Information (NCBI).

### Transcriptomics profiling analysis

To identify functionally related genes, particularly those involved in drought tolerance, gene ontology (GO) analysis was performed on the DEGs using the Search Tool for the Retrieval of Interacting Genes/Proteins (STRING). Subsequently, protein–protein interaction (PPI) networks were predicted.

### Statistical analysis

Biochemical studies were conducted in triplicate. The results from leaf sample analyses were subjected to one-way analysis of variance (ANOVA) and Tukey’s honestly significant difference (HSD) test at *p* < 0.05 to determine statistically significant differences between the mean values of each parameter. Statistical analyses were performed using SPSS software (Version 15.0, SPSS Inc., USA).

## Results and discussion

### Total soluble protein content

**Figure 1:**
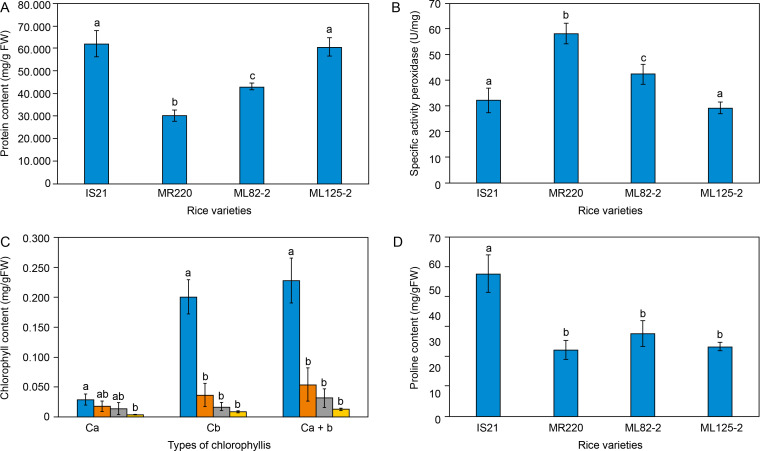
Biochemical analysis of the parent lines, IS21 and MR220, and mutant lines, ML 82-2 and ML 125-2 based on the (**A**) total soluble protein content, (**B**) specific peroxidases activity, (**C**) total chlorophyll content, and (**D**) proline content

The soluble protein biochemical assay showed variability among different *O. sativa* seedlings. As shown in Figure 1A, IS21 and ML 125-2 exhibited the highest total soluble protein content, measuring 61.84 ± 5.78 mg/g FW and 60.6 ± 3.92 mg/g FW, respectively. Padmavathi et al. (2013) reported that high total soluble protein content, primarily composed of amino acids, serves as osmolytes that maintain water uptake and turgidity in plant cells.

Referring to Figure 1A, the significantly higher soluble protein content in IS21 and ML 125-2 suggests their superior ability to maintain water uptake and turgidity compared to MR220 and ML 82-2, even under nondrought conditions. Soluble proteins, particularly enzymes, and stress-related proteins help protect cells from drought-induced oxidative stress by mitigating damage and repairing cellular structures (Rajput et al., 2021). For instance, late embryogenesis abundant (LEA) proteins are a large group of hydrophilic proteins synthesized in response to abiotic stresses. These proteins scavenge reactive oxygen species (ROS) and maintain cell turgor pressure by accumulating in the cytoplasm to absorb water (Aziz et al., 2021).

Consistent with the findings in Figure 1A, the higher soluble protein content in IS21 and ML 125-2 indicates their enhanced ability to mitigate oxidative stress-induced damage under drought conditions compared to MR220 and ML 82-2. In other words, a higher amount of LEA proteins and osmolytes are present in IS21 and ML 125-2 to scavenge ROS as compared to MR220 and ML 82-2. In addition, LEA proteins and heat shock proteins (HSPs) function as molecular chaperones, ensuring proper protein folding into stable three-dimensional structures and preventing protein misfolding and aggregation (Aziz et al., 2021; Haq et al., 2019). Thus, beyond ROS scavenging, IS21 and ML 125-2 exhibit greater drought tolerance due to the presence of HSPs, which help maintain protein conformation under stress.

The findings in the current study suggest that the accumulation of amino acids such as LEA proteins or HSPs leads to high soluble protein content that aids in ROS scavenging as well as correct protein folding under drought stress. The high soluble protein content thereby indicates a better drought tolerance capability of IS21 and ML 125-2. With this, the statistical analysis reported that ML125-2 has no significant difference with the IS21 parent line, specifying possible drought tolerance inheritance of the progeny from the IS21 parent line in terms of soluble protein content.

Among the rice lines analyzed, MR220 exhibited the lowest soluble protein content, measuring only 30.08 ± 2.64 mg/g FW (Figure 1A). Although MR220 serves as the parent line for ML 82-2 and ML 125-2, Figure 1A clearly distinguishes MR220 from its progeny in terms of total soluble protein content. The higher protein content in the daughter lines suggests enhanced drought tolerance and improved agronomic traits, likely resulting from backcrossing with IS21 and MR220.

To further justify the relationship between high total soluble protein content and drought tolerance in rice plants, Diniz et al. (2020) proposed that amino acid degradation supplies carbon skeletons to the tricarboxylic acid (TCA) cycle as an adaptive response to drought stress. Carbon skeletons in the TCA cycle play a crucial role in maintaining signaling and osmoregulation in plants under drought conditions (Diniz et al., 2020; Li et al., 2021). Understanding the significance of amino acids and their impact on drought resistance in plants is therefore essential.

In contrast with other rice lines, MR220, which exhibited the lowest soluble protein content, may be more prone to protein misfolding and osmotic pressure dysregulation due to the lack of HSPs and precursors for the TCA cycle. Consequently, MR220 is likely more susceptible to drought stress compared to its rice mutant lines, implying improved agronomic traits in the progenies as products of backcross breeding with IS21.

### Specific activity of peroxidase

Figure 1B illustrates the inverse association between specific peroxidase activity (SPA) and total soluble protein content. The peroxidase assay in this experiment exposed crude extracts of the samples to a relatively high concentration of hydrogen peroxide (30%), where guaiacol oxidation by guaiacol peroxidase (POD) eliminates hydrogen peroxide, preventing its detrimental effects on plants. Research has suggested that high concentrations of hydrogen peroxide can induce oxidative damage to biomolecules, leading to cell death (Cerny et al., 2018). Under unfavorable environmental conditions such as drought, increased hydrogen peroxide production can damage proteins, membrane lipids, and nucleic acids (Javed et al., 2021; Singh et al., 2017; Xie et al., 2019). Since the volume and concentration of hydrogen peroxide and guaiacol were controlled, the level of POD required to oxidize guaiacol served as the responding variable, denoted as SPA.

Findings in Figure 1B reveal an inverse association with the data in Figure 1A, where IS21 and ML 125-2 exhibited the lowest SPA levels, at 32.123 ± 4.835 U/mg and 29.213 ± 2.305 U/mg, respectively. These results suggest that a lower amount of POD was required to oxidize guaiacol and eliminate the high concentration of hydrogen peroxide, resulting in lower SPA values compared to ML 82-2 and MR220. This indicates that IS21 and ML 125-2 are better equipped to cope with abiotic stress.

In contrast to its total soluble protein content, MR220 recorded the highest SPA level among all rice varieties, requiring 58.285 ± 4.078 U/mg of POD enzymes to produce a 0.01 absorbance change per minute per unit of activity. In comparison, IS21 and ML 125-2 required only 32.123 ± 4.835 U/mg and 29.213 ± 2.305 U/mg, respectively, to catalyze guaiacol oxidation and eliminate the same amount of H_2_O_2_. In other words, under the same volume and concentration of H_2_O_2_, droughttolerant rice would be capable of eliminating the H_2_O_2_ with a minimal amount of POD used, which would result in a lesser SPA level. A high SPA level, as observed in MR220, suggests an inability to tolerate drought stress, necessitating a greater POD enzyme activity to mitigate oxidative stress. Statistical analysis revealed significant differences between MR220 and the mutant lines ML 82-2 and ML 125-2, further indicating that the mutant lines did not inherit the genotypic traits of peroxidases from MR220. Conversely, ML 125-2 showed no significant difference from IS21, suggesting improved agronomic traits and a probable inheritance of POD biochemical characteristics from its parent line, IS21.

Peroxidases are also known to strengthen plant cell walls by catalyzing cross-linking between cell wall components, specifically through H_2_O_2_-mediated oxidation of aromatic cell wall compounds (Francoz et al., 2015). A study on white clover leaves revealed that low water potential enhanced the activation of guaiacol peroxidase, which was closely associated with increased lignin content (Gall et al., 2015b). Further research has demonstrated that cross-linked phenolic compounds, such as lignin, serve as strong indicators of plant resistance to drought stress (Yang et al., 2006). When lignin is introduced, cell wall rigidification and growth arrest are triggered under drought stress, leading to reduced crop productivity as a resistance mechanism to water deficit conditions. The compact, tightly bound, and less permeable cell wall helps prevent water loss to the apoplast, functioning as a hydrophobic stabilizing property to maintain leaf turgor under low water potential (Hura et al., 2012, 2013). Therefore, MR220 appears to exhibit drought tolerance through peroxidation as a stress-responsive mechanism that regulates leaf stiffening under water-deficit conditions.

Figure 1B further highlights that ML 82-2 displayed an intermediate SPA of 42.506 ± 3.917 U/mg, suggesting better ROS scavenging and leaf stiffening performance compared to ML 125-2 and IS21 under drought stress, although it remained more susceptible than the MR220 rice cultivar. Beyond the established roles of peroxidases, studies have also emphasized their importance in stress-signaling pathways. Reis et al. (2022) concluded that H_2_O_2_ in the root system can enhance long-distance signaling in tomato plants, facilitating early stomatal closure as a coping mechanism to reduce transpiration under water-limited conditions. Further in-depth studies have linked ROS scavenging activity to abscisic acid (ABA) signaling pathways, in which ABA induces H_2_O_2_ synthesis in guard cells via membrane-bound nicotinamide adenine dinucleotide phosphate (NADPH) oxidase. H_2_O_2_ subsequently mediates stomatal closure by activating plasma membrane calcium ion channels through hyperpolarization (Carvalho, 2008). In relation to drought tolerance, peroxidases that scavenge H_2_O_2_ are believed to play an indirect role in stomatal regulation via ABA signaling pathways, helping to prevent excessive water loss under drought stress.

Overall, the statistical analysis highlighted significant differences between the rice lines in their ability to cope with drought conditions, with MR220 and ML 82-2 being more dependent on SPA for drought tolerance.

### Chlorophyll content

Figure 1C shows an overall higher C_b_ content than C_a_. Under normal conditions, C_a_, which is involved in oxygenic photosynthesis, serves as the primary pigment that absorbs light energy from violet-blue (420–450 nm) and orange-red (600–700 nm) wavelengths for photosynthesis (Landi et al., 2020). However, under limited light conditions, shorter blue wavelengths (400–570 nm) predominate (Hogewoning et al., 2012). Consistent with the findings in Figure 1C, the abundant supply of C_b_ may broaden the spectrum of absorbed light, enhancing the photosynthesis rate and ensuring sufficient adenosine triphosphate (ATP) production. A higher C_b_ content may thus serve as an indicator of a droughtcoping mechanism in rice lines.

Based on Figure 1C, IS21 exhibited an abnormally high total chlorophyll content of 0.2281 ± 0.0375 mg/g FW, while MR220, ML 82-2, and ML 125-2 displayed total chlorophyll contents of 0.054 ± 0.0279 mg/g FW, 0.0312 ± 0.0154 mg/g FW, and 0.0127 ± 0.0016 mg/g FW, respectively. While high chlorophyll content generally indicates an increased photosynthetic rate, which enhances plant performance under drought-stress conditions, it may also suggest pre-germination chlorophyll accumulation in dormant seeds. Nakajima et al. (2012) investigated chlorophyll retention in *Arabidopsis* seeds, where mutant lines failed to degrade chlorophyll properly. Consequently, a decline in seed germination rate after storage was observed, underscoring the importance of chlorophyll degradation in sustaining seed longevity. This finding further supports the idea that chlorophyll degradation is essential, as residual chlorophyll can reduce seed viability, shorten shelf life, and increase the risk of plant material contamination (Hu et al., 2023). Therefore, the excessive chlorophyll content in IS21 may result from incomplete chlorophyll degradation before entering dormancy.

Figure 1C also illustrates that MR220 exhibited a higher total chlorophyll content than both mutant lines, although it remained significantly lower than IS21. Under water deficit and elevated temperature conditions, chlorophyll content declines due to the disruption of thylakoid membrane integrity caused by heat stress (Dias et al., 2009; Hanif et al., 2021a), as well as disturbances in electron transport within the photosynthetic machinery (Adeel et al., 2017; Hanif et al., 2021; Mathur et al., 2014). According to Gujjar et al. (2020), the photosynthesis rate in rice plants is severely affected by water scarcity, leading to increased leaf wilting, reduced yield, and decreased fresh biomass. These findings underscore the critical role of chlorophyll in sustaining photosynthesis, particularly under drought conditions (Nounjan et al., 2020). Accordingly, MR220 demonstrated better adaptation and photosynthetic capability than ML 82-2 and ML 125-2 under drought stress.

However, statistical analysis revealed no significant difference in total chlorophyll content between MR220 and the mutant lines, suggesting that ML 82-2 and ML 125-2 possess a similar level of drought adaptability as MR220. Conversely, MR220, ML 82-2, and ML 125-2 appear to be more susceptible to drought stress than IS21 due to their lower photosynthetic rates.

Beyond dormancy, the pronounced difference in chlorophyll content observed in IS21 is inferred to be a stress adaptation mechanism. Unlike other rice lines that utilize chlorophyll for stomatal regulation and the prevention of excessive water loss, IS21 primarily relies on chlorophyll for photosynthesis. Feller et al. (2016) and Lamaoui et al. (2018) emphasized the importance of stomatal closure in reducing photosynthesis and transpiration rates, thereby enhancing water-use efficiency and improving acclimatization to water-deficit conditions.

### Proline content

The proline content of *O. sativa* seedlings was determined after two weeks of germination. As shown in Figure 1D, IS21 exhibited the highest proline content at 0.0477 ± 0.0063 mg/g FW, with statistical analysis indicating a significant difference compared to other rice varieties. Dien et al. (2019) reported that drought-tolerant rice varieties accumulate proline as an osmoprotectant under water-deficit conditions, with levels rapidly degrading upon rewatering. Environmental stresses stimulate the production of ROS as part of the stress response mechanism, which can cause damage to membrane components and disrupt signal transduction pathways. In this context, proline functions as an antioxidant, scavenging ROS and acting as a singlet oxygen quencher to alleviate drought stress (Hayat et al., 2012). Thus, higher proline content indicates a stronger ROS scavenging capacity in rice cultivars when subjected to drought stress. Accordingly, the elevated proline levels in IS21 suggest superior ROS scavenging performance and a more effective drought stress response compared to other rice varieties.

As depicted in Figure 1D, MR220 exhibited the lowest proline content at 0.0221 mg/g FW, indicating greater susceptibility to drought stress relative to the other rice varieties. Proline, also known as a molecular chaperone, is synthesized in response to environmental fluctuations to facilitate temperature adaptation and rapid recovery following heat or cold stress (Ghosh et al., 2022). Since drought conditions can lead to protein misfolding, stress-induced genes, such as those encoding chaperone proteins, play a critical role in maintaining protein conformation (Ghosh et al., 2022; Yang et al., 2010). Studies have shown that overexpression of soybean luminal binding protein, a member of the HSP70 protein chaperone family, confers drought resistance in soybean plants by delaying drought-induced leaf senescence. Consistent with this, proline accumulation in rice plants protects against drought stress, suggesting that MR220 may have a lower abundance of chaperones, making it less equipped to withstand abiotic stresses.

Statistical analysis revealed that MR220 has no significant difference with the mutant lines, implying that both the mutant lines also comprise insubstantial chaperone quantity. Therefore, MR220, along with ML 82-2 and ML 125-2 are less tolerant to drought stress in comparison to IS21. Under normal conditions, the proline content in ML 82-2 and ML 125-2 was recorded at 0.0276 mg/g FW and 0.0232 mg/g FW, respectively, as shown in Figure 1D. According to Bunnag et al. (2013), rice cultivars subjected to drought stress exhibited higher proline levels (> 0.05 mg/g FW), confirming that proline accumulation predominantly occurs in plants under drought conditions. Similar findings were reported by Mishra et al. (2018), who observed elevated proline accumulation in water-deficit conditions rather than in well-watered rice varieties. This phenomenon can be attributed to the enhancement of substomatal carbon dioxide levels, stomatal conductance, and the maintenance of leaf turgidity in response to drought stress (Ghosh et al., 2022). As proline facilitates osmotic adjustment and stabilizes cellular structures, its accumulation is often an indicator of leaf dehydration and has been correlated with plant stress susceptibility (Dien et al., 2019). For context, proline content in ML 82-2 and ML 125-2 in the present study can only suggest that these mutant lines have a certain extent of drought tolerance, but further research on these mutant lines under drought treatment should be conducted to justify its tolerance towards drought stress.

### Transcriptomics profiling analysis

**Figure 2:**
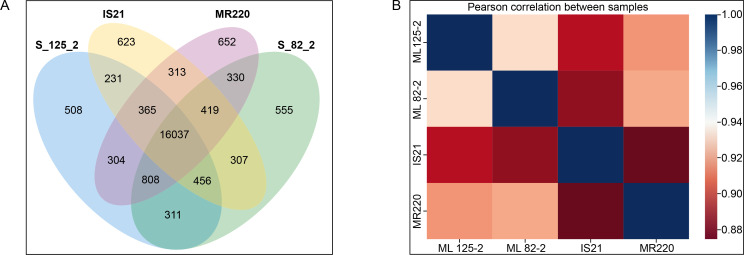
Transcriptomics profiling analysis of the rice cultivars with (**A**) Venn diagram displaying the gene count for each cultivar and (**B**) correlation heatmap comparing both mutant lines and parent lines

For the transcriptomics profiling analysis, the correlation between parent and mutant lines was assessed using a Venn diagram of co-expressed genes (Figure 2A) and a correlation heatmap (Figure 2B). As shown in Figure 2A, ML 82-2 and ML 125-2 shared 808 genes with the parent line MR220 but only 456 genes with IS21. These results suggest that the progeny exhibit a closer genetic alignment with MR220, demonstrating a higher genotypic resemblance to this parent line.

Consistent with Figure 2A, the Pearson correlation heatmap in Figure 2B further supports these findings. The data indicate that ML 82-2 is more closely correlated with MR220 than with IS21. Similarly, ML 125-2 showed a higher genetic association with MR220, surpassing IS21 by 2.1%. Additionally, ML 125-2 exhibited a stronger connection with IS21 than ML 82-2 when comparing the two mutant lines.

### Gene ontology

#### Osmolytic regulation

To complement the biochemical tests conducted, transcriptomic data analysis was performed across all rice lines to further validate and verify the presence of drought-resistance-related genes in the mutant lines. Osmoregulation through osmolytes, such as amino acids, plays a crucial role in facilitating water uptake and maintaining plant turgidity under drought stress.

For the CC category in the GO analysis of ML 125-2 vs. IS21, the overrepresented functional annotation was the extracellular region. The transcriptomic profile identified *substandard starch grain4* (*SSG4*) as one of the enriched genes. Generally, *SSG4* is responsible for forming starch granules, which are essential for plastid maintenance and cellular function (Matsushima et al., 2014). Similar to the downregulation of glycosyl hydrolase (*GH*) in ML 82-2 and MR220, *SSG4* is critical for starch production and storage. The downregulation of *SSG4* potentially compromises drought tolerance in rice cultivars due to reduced starch availability, which is necessary for maintaining water balance, photosynthesis, and other metabolic activities under drought stress (Pfister et al., 2016).

According to Seleiman et al. (2021), stored starch mobilization occurs during drought stress to provide energy in the form of glucose, supporting energy production, osmotic adjustment, and turgor pressure maintenance in plant cells. In this context, the downregulation of *SSG4* is speculated to be a drought-stress response, prioritizing survival over development in rice. Several studies further support the idea that resource reallocation from starch synthesis serves as an adaptive mechanism to conserve energy under drought conditions (Du et al., 2020; Seleiman et al., 2021).

Thus, the mutant lines ML 125-2 and ML 82-2 appear to exhibit drought tolerance through trade-off mechanisms, where the downregulation of *SSG4* and *GH* reallocates starch hydrolysis resources for downstream osmotic adjustments. This also suggests the inheritance of drought-adaptive genes from the parent lines IS21 and MR220. These findings align with the biochemical results in Figure 1A, which showed the upregulation of amino acids involved in osmoregulation, facilitating water uptake and maintaining plant turgidity.

A highly annotated GO category in ML 82-2 and MR220 is the CC category, with the extracellular region remaining highly significant. In general, plant proteases play a crucial role in drought response signaling pathways, regulating mechanisms such as stomatal development, stomatal aperture adjustment, ROS production, and amino acid remobilization (D’Ippólito et al., 2021).

This is supported by the upregulation of the *Serpin-Z1* (*OrysaZ1*) gene in ML 82-2. Studies have identified serpins, also known as serine protease inhibitors, as stressinduced negative regulators of cell death in plant immune systems (Rehman et al., 2020). The mechanism involves the inactivation of target proteinases through large conformational changes and the formation of kinetically stable covalent complexes with target enzymes (Hajibarat et al., 2022). Accordingly, ML 82-2 appears to confer drought tolerance through the upregulation of *OrysaZ1*, modulating proteolysis activity under water-deficit stress – an adaptive trait predominantly inherited from its parent line, MR220. These findings also support the biochemical results in Figure 1, emphasizing the importance of osmotic adjustment for stomatal conductance.

Regarding stomatal conductance, *RGA* genes have been implicated in facilitating stomatal closure via ABA signaling pathways, thereby reducing water loss under drought conditions. Notably, ADP binding was identified as the most significantly enriched MF GO term in ML 82-2, ML 125-2, and IS21, where the functionally enriched *RGA* genes (*RGA3, RGA5,* and *RGA1.1-4*) were detected. These findings align with the data in Figure 1B, demonstrating that ABA signaling pathways played a role in the 30% hydrogen peroxide drought treatment, preventing excessive water loss in rice plants. The results further underscore the inheritance of *RGA* genes by the mutant lines from their parent line, IS21.

#### Stress proteins and protein protection

In addition to osmotic adjustments and stomatal conductance, stress proteins that provide protein protection play a crucial role in ensuring proper drought stress responses in plants under water-deficit conditions. Research has shown that drought-stressed rice plants are also at risk of fungal pathogen attacks, which further compromise their ability to withstand droughtinduced stress (Ahluwalia et al., 2021).

Notably, resistance gene analogs (RGA) contain conserved domains and motifs that primarily function in pathogen resistance (Cortaga et al., 2022; Sekhwal et al., 2015). The presence of *RGA* genes in the ADP binding GO term within the MF category for both mutant lines and IS21 suggests that these rice lines possess some degree of pathogen resistance in addition to drought tolerance. The nucleotide-binding site–leucine-rich repeat receptor proteins in *RGA* genes act as signaling components that interact with pathogen receptors to form a resistome complex, ultimately triggering apoptosis by destabilizing cell membrane integrity (Adachi et al., 2019; Ijaz et al., 2022; Shi et al., 2020; Xiong et al., 2020).

The presence of pathogen-resistance-related *RGA* genes of RGA in ML 82-2 and ML 125-2 further implies the protection of plant and protein structures from pathogens. The findings denote the ability and importance of mutant lines to confer disease resistance and protect other drought-tolerant related proteins from pathogen attack.

Interestingly, a significant GO term for MF in the ML 82-2, ML 125-2, and MR220 is chitinase activity, where pathogenesis-related chitinase proteins primarily function as a defense against pathogens. Chitinases such as *C10150, C10122, C10923, CHT3, CHT4, CHT6,* and *CHT9* play essential roles in embryogenesis, defense against fungal pathogens, and abiotic stress responses in plants. However, the precise mechanisms by which chitinases confer drought tolerance are yet to be fully elucidated (Sakamoto et al., 2017; Thapa et al., 2023).

Lv et al. (2022) conducted a study on soybean (*Glycine max*), where qRT-PCR analysis revealed an upregulation of chitinase genes at different time intervals of drought, confirming their association with plant stress responses. Similarly, a significant increase in chitinase gene expression was observed in *Arabidopsis* (Cho et al., 2013; Liu et al., 2020), *Capsicum* (Mishra et al., 2021), Bermuda grass (Nakamura et al., 2008), and *Catharanthus roseus* (Ali et al., 2021) in response to drought tolerance, suggesting a strong link between chitinases and drought stress response mechanisms in plants. These findings highlight the significance of chitinase genes in the mutant lines and suggest that this trait was inherited from the parent line MR220.

Bowman-Birk inhibitors (BBIs), such as *BBI3-3* and *BBI2-3*, were also identified as significant genes in the CC category for all rice lines, particularly in the extracellular region (Table 1). BBIs are trypsin inhibitors that are elevated during injury or abiotic stress to suppress drought-induced oxidative stress (Domagalska et al., 2020). Supporting this, Dhanushokdi et al. (2018) found that hydrogen peroxide production during drought stress enhances proteolysis activity, where elevated BBI levels contribute to leaf senescence and drought stress sensitivity.

**Table 1:** Top 10 most significant enriched GO annotations and the respective differentially expressed genes (DEGs) in all rice lines

Lines	Category	Description	p-value	Gene Symbol
ML 125-2 vs. IS21	CC	Extracellular region	3.1E-11	C10150/RIXI/POX8/OXO1/C10122/BGLU6/EXB15/ SSG4/BBI3-3/DIP3/ PRX30/EXPA32/PR1-51/RAG2/FOR1/PPC2B/CP1/ WDA1/CKX2
	MF	ADP binding	2.6E-10	PI63/OSWD40-115/WX1/PIZ/PGK4/PITA/WRKY41/ WRKY125/PI9/ BPH26/rNBS41/LSSR1/RLS1/PISH/PGK2/RGA5/ RPR1/VQ34
	BP	RNA modification	7.4E-10	WAF1/ORRM1/SPED1/EMP5/NCS6/IPT9/PPR1
	MF	Heme binding	1.6E-06	POX8/HB1/HB2/SCP28/D2/APX2/KO1/ABA8OX3/HB3/KO4/PRX32/ PRX45/AOS3/POX22/PRX48/PRX12/ CYP86A7-2/F3’H/PRX34/CYP71Z4/ SD37/PRX19/D11/CYP76M5/CYP99A3/PRX98/PRX88/ PRXA/PER4/APX3/F5H1/CYP734A4/NR2/HPL3/PSR1/ D35/SL/CATC/C4H2/PRX17/PRX49
ML 125-2 vs. MR220	MF	Chitinase activity	2.4E-07	C10150/GH/C10122/XIP/RIXI/CHT9/C10923/ CHT3/CHT6
ML 82-2 vs. IS21	MF	ADP binding	1.2E-08	RGA3/WRKY41/rNBS17/PISH/WX1/PITA/PIZT/PGK4/ WRKY125/OSWD40-115/BPH26/WRKY123/YR18/ PGK2/RGAA1.1-4/PI3/PID3
	BP	RNA modification	5.0E-07	WAF1/PLS13/ORRM1/OSSTA84/SPED1/IPT9/NCS6/ OSDJC63/SIP2
ML 82-2 vs. MR220	BP	Xylan catabolic process	3.8E-07	C10150/RIXI/C10923
	MF	Chitinase activity	5.5E-07	C10150/GH/C10122/XIP/RIXI/C10923/CHT9/CHT4
	CC	Extracellular region	1.6E-06	C10150/GH/PRX61/C10122/XIP/ORAP1/RIXI/PR1/ PRX109/OLP1/C10923/DRR206/PRX63/GLP8-6/ PRX83/ORYSAZ2B/GLP24/LAC14/PRX43/PRX95/ SAG12/ MAN5/PRX2/EXLA4/YY1/PRX98/PRX69/GLP1/PR5/ XTH19/GLP8-13/LAC24/ PMEI25/PRX42/PRX130/BBI3-3/PRX66/BBI2-3/XTH6/ LAC5/PRX8.1/GRP2

CC – cellular component, MF – molecular function, BP – biological process

Under drought stress, protein breakdown is facilitated by proteases such as cysteine, asparagine, and serine proteases (Malefo et al., 2020). Since BBIs act as serine protease inhibitors, they catalyze the hydrolysis of specific peptide bonds in substrates containing serine amino acid residues in their active sites. Proteolysis is a key adaptation mechanism in plants under abiotic stress, as the degradation of misfolded proteins generates amino acids for the synthesis of new proteins (Gur et al., 2011).

Thus, the mutant lines demonstrate abiotic stress resistance through protein breakdown regulated by BBI genes, a trait inherited from their parent lines. These findings align with the data presented in Figure 1, supporting the role of stress proteins such as trypsin inhibitors in protecting plants by degrading misfolded proteins under drought stress.

### Antioxidant defense

Peroxidases were detected across all rice cultivars, including POX8, POX22, PRXA, PRX2, PRX12, PRX17, PRX19, PRX30, PRX32, PRX34, PRX43, PRX45, PRX48, PRX49, PRX61, PRX63, PRX66, PRX69, PRX83, PRX88, PRX95, PRX98, PRX103, and PRX109. These findings support the data in Figure 1B, which emphasizes the importance of peroxidases in ROS scavenging activities.

While MR220 carried out the highest SPA as compared to other rice cultivars, data in Table 1 suggests the presence of peroxidases-related genes in all rice cultivars, implying the dependence of MR220 on peroxidases to confer drought tolerance as compared to other rice cultivars.

### Photosynthesis

A significantly enriched GO term in the heme binding category of the MF classification in ML 125-2 and IS21 includes dwarf-related genes, such as *dwarf ebisu* (*D2*)*, dwarf shinkaneaikoku* (*D11*)*,* and *semidwarf 37* (*SD37*). Previous studies have associated *d2* with shorter culm, leaf blade, and sheath length, as well as higher chlorophyll content in *O. sativa*, which contributes to improved water-use efficiency (Priatama et al., 2022). These findings suggest that ML 125-2 and IS21 exhibit drought tolerance through dwarfism traits and enhanced chlorophyll content as regulated by these genes.

This implies that ML 125-2, having inherited dwarfrelated genes from IS21, should have exhibited similar chlorophyll content. However, the contradicting findings in Figure 1C, where IS21 displayed an abnormally high chlorophyll content, can be attributed to seed dormancy and incomplete chlorophyll degradation before germination.

This is further supported by the presence of WRKY41 genes, primarily detected in IS21. Research by Ding et al. (2014) proposed that WRKY41 interacts with ABA to regulate abscisic acid insensitive 3 (ABI3), which plays a role in seed dormancy. Additionally, a study by Duan et al. (2017) on *Arabidopsis thaliana* linked WRKY41 to the positive regulation of ABI3, further confirming its role in seed dormancy.

In summary, dwarf-related genes not only provide mechanical support but also enhance chlorophyll content, contributing to drought tolerance. The presence of *WRKY41* in the transcriptomic profile of IS21 had also proven the dormancy of seeds.

### Mechanical support

In both mutant lines and IS21, WAVY LEAF 1 (WAF1) genes were detected under the RNA modification GO term in the BP category. A study by Abe et al. (2010) suggested that *WAF1* stabilizes ta-siRNA, which is essential for shoot development and shoot apical meristem maintenance. In general, shoots provide mechanical support to plants while also serving as water and nutrient storage structures. This suggests that the mutant lines inherited structural-maintaining genes from IS21, enabling water and nutrient storage in rice plants to better withstand drought conditions.

Additionally, Wang et al. (2019) reported that *WAF1* participates in the small RNA pathway, influencing the polarity of lemma and palea. Lemma and palea are key floral organs that determine grain length and shape, which are indirectly linked to water-deficit stress responses by reducing excessive water loss. Thus, a higher expression of *WAF1* is associated with improved drought tolerance, as it supports shoot development for sufficient water and nutrient storage while regulating lemma and palea formation. Therefore, ML 82-2 and ML 125-2 possess the drought-tolerance-related gene *WAF1*, ensuring nutrient and water storage in rice plants under drought conditions—a trait inherited from IS21.

A high enrichment of chitinase activity in the MF category was identified in ML 82-2, ML 125-2, and MR220. Additionally, the xylan catabolic process was significantly enriched in the BP category, while the extracellular region was enriched in the CC category in ML 82-2 and MR220 (Table 1). The DEGs common to these rice lines include xylanase inhibitor protein (XIP) and rice xylanase inhibitor (RIXI). Under drought stress, stress-responsive cis-acting elements in the promoter regions of xylanase inhibitors play a role in drought adaptation mechanisms (Dornez et al., 2010; Liu et al., 2021). Cis-acting elements serve as binding sites for transcription factors, regulating gene expression in signal transduction pathways (Mahmood et al., 2020). In drought-tolerant plants, drought-responsive transcription factors, such as dehydration-responsive element-binding proteins, bind to dehydration-responsive element/C-repeat motifs in the promoter regions of xylanase inhibitor proteins. Activation of the bound complex is initiated under drought conditions, resulting in an upregulation of the xylanase inhibitor genes to further maintain the cell wall integrity of plants (Wang et al., 2022). The presence of XIPs and RIXIs in ML 82-2 and ML 125-2 suggests the inheritance of xylanase inhibitor genes from MR220, contributing to the maintenance of cell wall integrity under drought stress.

The genomic profiles of both ML 125-2 and IS21 revealed Wax-Deficient Anther 1 (WDA1) as a DEG within the extracellular region in the GO enrichment analysis. Under water-limited conditions, as stomata close, transpiration still occurs through nanoscale diffusion, crossing the cuticle of leaf tissues in vegetative plants (Bi et al., 2017; Jung et al., 2006).

A thicker cuticle helps retain water by reducing transpiration rates, thereby improving drought tolerance (Bennett et al., 2012). Studies have linked the *WDA1* gene to the biosynthesis of very long-chain fatty acids, which serve as monomers for cutin, a vital component of cuticles that prevents uncontrolled water loss (Jung et al., 2006; Zhu et al., 2013).

Thus, the upregulation of *WDA1* genes likely contributes to the formation of thicker cuticles via wax biosynthesis, reducing water loss in plants. In short, *WDA1* genes in the extracellular regions of ML 125-2 and IS21 function as a drought stress response mechanism, enhancing cuticle thickness to minimize transpiration-induced water loss.

## Conclusions

The biochemical parameters that exhibited significant differences between the mutant lines and parent lines were total soluble protein content and SPA. The mutant lines demonstrated higher or improved biochemical content, with ML 82-2 being more dependent on specific peroxidase activity, whereas ML 125-2 relied more on total soluble protein content for drought tolerance. Additionally, several drought-tolerance-related genes were identified based on their functions. These included *SSG4, GH, OrysaZ1, RGA*, chitinase genes, peroxidase-related genes, *BBIs*, dwarf-related genes, and *WRKY41*, all of which contribute to biochemical support and were inherited by ML 82-2 and ML 125-2 from their parent lines. Furthermore, transcriptomic profiling analysis highlighted the importance of mechanical support in drought-resistant plants, with *WDA1, WAF1, RIXI,* and *XIP* playing key roles in drought adaptation mechanisms.

## References

[cit0001] Abe M., Yoshikawa T., Nosaka M., Sakakibara H., Sato Y., Nagato Y., Itoh J.I. (2010) Wavy Leaf1, an ortholog of Arabidopsis Hen1, regulates shoot development by maintaining microRNA and trans-acting small interfering RNA accumulation in rice. Plant Physiol. 154(3): 1335–46. DOI: 10.1104/Pp.110.160234. 20805329 PMC2971610

[cit0002] Adachi H., Contreras M., Harant A., Wu C.H., Derevnina L., Sakai T., Duggan C., Moratto E., Bozkurt T.O., Maqbool A., Win J., Kamoun S. (2019) An N-terminal motif in NLR immune receptors is functionally conserved across distantly related plant species. Elife 8. DOI: 10.7554/Elife.49956. PMC6944444 31774397

[cit0003] Ahluwalia O., Singh C.P., Bhatia R. (2021) A review on drought stress in plants: Implications, mitigation and the role of plant growth-promoting rhizobacteria. Resour. Environ. Sustain. 5: 100032.

[cit0004] Aleem M., Riaz A., Raza Q., Aleem M., Aslam M., Kong K., Atif R.M., Kashif M., Bhat J.A., Zhao T. (2022) Genomewide characterization and functional analysis of Class III peroxidase gene family in soybean reveal regulatory roles of GsPOD40 in drought tolerance. Genomics 114(1): 45–60. DOI: 10.1016/J.Ygeno.2021.11.016. 34813918

[cit0005] Ali E.F., El-Shehawi A.M., Ibrahim O.H.M., Abdul-Hafeez E.Y., Moussa M.M., Hassan F.A.S. (2021) A vital role of chitosan nanoparticles in improvisation of drought stress tolerance in Catharanthus roseus (L.) through biochemical and gene expression modulation. Plant Physiol. Biochem. 161: 166–175. DOI: 10.1016/J.Plaphy.2021.02.008. 33610861

[cit0006] Allagulova C., Avalbaev A., Fedorova K., Shakirova F. (2020) Methyl jasmonate alleviates water stress-induced damages by promoting dehydrins accumulation in wheat plants. Plant Physiol. Biochem. 155: 676–682. DOI: 10.1016/J.Plaphy.2020.07.012. 32861034

[cit0007] Almagro L., Gómez Ros L.V., Belchi-Navarro S., Bru R., Ros Barceló A., Pedreño M.A. (2009) Class III peroxidases in plant defence reactions. J. Exp. Bot. 60(2): 377–390. DOI: 10.1093/Jxb/Ern277. 19073963

[cit0008] Anon. (2021) Making agrifood systems more resilient to shocks and stresses food and agriculture. DOI: 10.4060/Cb4476en.

[cit0009] Anon. (n.d.-a) El Nino hits rice industry | The Star. Retrieved November 3, 2023. [Online]. Available: https://www.the-star.com.my/news/nation/2023/07/08/el-nino-hits-rice-industry.

[cit0010] Anon. (n.d.-b) How India’s ban on some rice exports is ricocheting around the world | Business and Economy News | Al Jazeera. Retrieved November 3, 2023. [Online]. Available: https://www.aljazeera.com/economy/2023/8/16/how-indias-ban-on-some-rice-exports-is-ricocheting-around-the-world.

[cit0011] Anon. (n.d.-e) Rice planters will lose income if padi land converted for industry | The Star. Retrieved November 3, 2023. [Online]. Available: https://www.thestar.com.my/news/nation/2023/05/24/rice-planters-will-lose-income-if-padi-land-converted-for-industry.

[cit0012] Anon. (n.d.-f) Southeast Asia’s mounting food insecurity — Benarnews. Retrieved November 3, 2023. [Online]. Available: https://www.benarnews.org/english/commentaries/food-insecurity-southeast-asia-10162023151618.html.

[cit0013] Anon. (n.d.-g) UN report: Global hunger numbers rose to as many as 828 million in 2021. Retrieved November 3, 2023. [Online]. Available: https://www.who.int/news/item/06-07-2022-un-report--global-hunger-numbers-rose-to-as-many-as-828-million-in-2021.

[cit0014] Anon. (n.d.-h) What is mutation breeding? | IAEA. Retrieved July 5, 2023. [Online]. Available: https://www.iaea.org/newscenter/news/what-is-mutation-breeding.

[cit0015] Aziz M.A., Sabeem M., Mullath S.K., Brini F., Masmoudi K. (2021) Plant Group II LEA proteins: Intrinsically disordered structure for multiple functions in response to environmental stresses. Biomolecules 11(11). DOI: 10.3390/Biom11111662. PMC8615533 34827660

[cit0016] Baadu R., Chong K.P., Gansau J.A., Zin M.R.M., Dayou J. (2023) A systematic review on physical mutagens in rice breeding in Southeast Asia. PeerJ 11: 1–28. DOI: 10.7717/Peerj.15682. PMC10590103 37868055

[cit0017] Bashir K., Hanada K., Shimizu M., Seki M., Nakanishi H., Nishizawa N.K. (2014) Transcriptomic analysis of rice in response to iron deficiency and excess. Rice 7(1): 1–15. DOI: 10.1186/S12284-014-0018-1. 26224551 PMC4884027

[cit0018] Bates L.S., Waldren R.P., Teare I.D. (1973) Rapid determination of free proline for water-stress studies. Plant Soil 39(1): 205–207. DOI: 10.1007/Bf00018060.

[cit0019] Bennett D., Izanloo A., Edwards J., Kuchel H., Chalmers K., Tester M., Reynolds M., Schnurbusch T., Langridge P. (2012) Identification of novel quantitative trait loci for days to ear emergence and flag leaf glaucousness in a bread wheat (Triticum aestivum L.) population adapted to Southern Australian conditions. Theor. Appl. Genet. 124(4): 697–711. DOI: 10.1007/S00122-011-1740-3. 22045047

[cit0020] Bi H., Kovalchuk N., Langridge P., Tricker P.J., Lopato S., Borisjuk N. (2017) The impact of drought on wheat leaf cuticle properties. BMC Plant Biol. 17(1): 1–13. DOI: 10.1186/S12870-017-1033-3. 28482800 PMC5422891

[cit0021] Bienert G.P., Chaumont F. (2014) Aquaporin-facilitated transmembrane diffusion of hydrogen peroxide. Biochim. Biophys. Acta Gen. Subj. 1840(5): 1596–1604. DOI: 10.1016/J.Bbagen.2013.09.017. 24060746

[cit0022] Bin Rahman A.N.M.R., Zhang J. (2023) Trends in rice research: 2030 and beyond. Food Energy Secur. 12(2): e390. DOI: 10.1002/Fes3.390.

[cit0023] Bradford M.M. (1976) A rapid and sensitive method for the quantitation of microgram quantities of protein utilizing the principle of protein-dye binding. Anal. Biochem. 72(1–2): 248–254. DOI: 10.1016/0003-2697(76)90527-3. 942051

[cit0024] Bunnag S., Pongthai P. (2013) Selection of rice (Oryza sativa L.) cultivars tolerant to drought stress at the vegetative stage under field conditions. Am. J. Plant Sci. 4(9): 1701–1708. DOI: 10.4236/Ajps.2013.49207.

[cit0025] Èerný M., Habánová H., Berka M., Luklová M., Brzobohatý B. (2018) Hydrogen peroxide: Its role in plant biology and cross-talk with signalling networks. Int. J. Mol. Sci. 19(9): 2812. 30231521 10.3390/ijms19092812 PMC6163176

[cit0026] Chen W., Gao Y., Xie W., Gong L., Lu K., Wang W., Li Y., Liu X., Zhang H., Dong H., Zhang W., Zhang L., Yu S., Wang G., Lian X., Luo J. (2014) Genome-wide association analyses provide genetic and biochemical insights into natural variation in rice metabolism. Nat. Genet. 46(7): 714–721. DOI: 10.1038/Ng.3007. 24908251

[cit0027] Cortaga C.Q., Latina R.A., Habunal R.R., Lantican D.V. (2022) Identification and characterization of genome-wide resistance gene analogs (RGAs) of durian (Durio zibethinus L.). J. Genet. Eng. Biotechnol. 20(1): 1–11. DOI: 10.1186/S43141-022-00313-8. 35157163 PMC8844316

[cit0028] Cruz De Carvalho M.H. (2008) Drought stress and reactive oxygen species: Production, scavenging and signaling. Plant Signal. Behav. 3(3): 156. DOI: 10.4161/Psb.3.3.5536. 19513210 PMC2634109

[cit0029] Dhanushkodi R., Matthew C., McManus M.T., Dijkwel P.P. (2018) Drought-induced senescence of Medicago truncatula nodules involves serpin and ferritin to control proteolytic activity and iron levels. New Phytol. 220(1): 196–208. DOI: 10.1111/Nph.15298. 29974467

[cit0030] Dias A.S., Lidon F.C. (2009) Evaluation of grain filling rate and duration in bread and durum wheat, under heat stress after anthesis. J. Agron. Crop Sci. 195(2): 137–147. DOI: 10.1111/J.1439-037x.2008.00347.X.

[cit0031] Dien D.C., Mochizuki T., Yamakawa T. (2019) Effect of various drought stresses and subsequent recovery on proline, total soluble sugar and starch metabolisms in rice (Oryza sativa L.) varieties. Plant Prod. Sci. 22(4): 530–545. DOI: 10.1080/1343943x.2019.1647787.

[cit0032] Ding Z.J., Yan J.Y., Li G.X., Wu Z.C., Zhang S.Q., Zheng S.J. (2014) WRKY41 controls Arabidopsis seed dormancy via direct regulation of ABI3 transcript levels not downstream of ABA. Plant J. 79(5): 810–823. 24946881 10.1111/tpj.12597

[cit0033] Diniz A.L., Da Silva D.I.R., Lembke C.G., Costa M.D.B.L., Ten-Caten F., Li F., Vilela R.D., Menossi M., Ware D., Endres L., Souza G.M. (2020) Amino acid and carbohydrate metabolism are coordinated to maintain energetic balance during drought in sugarcane. Int. J. Mol. Sci. 21(23): 9124. DOI: 10.3390/Ijms21239124. 33266228 PMC7729667

[cit0034] D’ippólito S., Rey-Burusco M.F., Feingold S.E., Guevara M.G. (2021) Role of proteases in the response of plants to drought. Plant Physiol. Biochem. 168: 1–9. DOI: 10.1016/J.Plaphy.2021.09.038. 34607206

[cit0035] Dorairaj D., Govender N.T. (2023) Rice and paddy industry in Malaysia: Governance and policies, research trends, technology adoption and resilience. Front. Sustain. Food Syst. 7: 1093605. DOI: 10.3389/Fsufs.2023.1093605.

[cit0036] Dornez E., Croes E., Gebruers K., De Coninck B., Cammue B.P.A., Delcour J.A., Courtin C.M. (2010) Accumulated evidence substantiates a role for three classes of wheat xylanase inhibitors in plant defense. Crit. Rev. Plant Sci. 29(4): 244–264. DOI: 10.1080/07352689.2010.487780.

[cit0037] Du Y., Zhao Q., Chen L., Yao X., Zhang W., Zhang B., Xie F. (2020) Effect of drought stress on sugar metabolism in leaves and roots of soybean seedlings. Plant Physiol. Biochem. 146: 1–12. DOI: 10.1016/J.Plaphy.2019.11.003. 31710920

[cit0038] El-Mouhamady A.B.A., Gad A.A.M., Abdel Karim G.S.A. (2022) Improvement of drought tolerance in rice using Line × Tester mating design and biochemical molecular markers. Bull. Natl. Res. Cent. 46(1): 1–20. DOI: 10.1186/S42269-021-00656-1.

[cit0039] Food and Agriculture Organization of the United Nations. (2023) Food Outlook: Biannual report on global food markets. FAO Rome 1(3): 3.

[cit0040] Francoz E., Ranocha P., Nguyen-Kim H., Jamet E., Burlat V., Dunand C. (2015) Roles of cell wall peroxidases in plant development. Phytochemistry 112(1): 15–21. DOI: 10.1016/J.Phytochem.2014.07.020. 25109234

[cit0041] Ghosh U.K., Islam M.N., Siddiqui M.N., Cao X., Khan M.A.R. (2022) Proline, a multifaceted signalling molecule in plant responses to abiotic stress: Understanding the physiological mechanisms. Plant Biol. (Stuttgart) 24(2): 227–239. DOI: 10.1111/Plb.13363. 34796604

[cit0042] Gitlin-Domagalska A., Maciejewska A., Dębowski D. (2020) Bowman-Birk inhibitors: Insights into family of multifunctional proteins and peptides with potential therapeutical applications. Pharmaceuticals 13(12): 1–40. DOI: 10.3390/Ph13120421. PMC7760496 33255583

[cit0043] Gujjar R.S., Banyen P., Chuekong W., Worakan P., Roytrakul S., Supaibulwatana K. (2020) A synthetic cytokinin improves photosynthesis in rice under drought stress by modulating the abundance of proteins related to stomatal conductance, chlorophyll contents, and Rubisco activity. Plants 9(9): 1106. DOI: 10.3390/Plants9091106. 32867335 PMC7569833

[cit0044] Gur E., Biran D., Ron E.Z. (2011) Regulated proteolysis in Gram-negative bacteria — how and when? Nat. Rev. Microbiol. 9(12): 839–848. DOI: 10.1038/Nrmicro2669. 22020261

[cit0045] Hajibarat Z., Saidi A. (2022) Senescence-associated proteins and nitrogen remobilization in grain filling under drought stress condition. J. Genet. Eng. Biotechnol. 20(1): 101. DOI: 10.1186/S43141-022-00378-5. 35819732 PMC9276853

[cit0046] Hanif S., Saleem M.F., Sarwar M., Irshad M., Shakoor A., Wahid M.A., Khan H.Z. (2021a) Biochemically triggered heat and drought stress tolerance in rice by proline application. J. Plant Growth Regul. 40(1): 305–12. DOI: 10.1007/S00344-020-10095-3.

[cit0047] Hanif S., Saleem M.F., Sarwar M., Irshad M., Shakoor A., Wahid M.A., Khan H.Z. (2021b) Biochemically triggered heat and drought stress tolerance in rice by proline application. J. Plant Growth Regul. 40(1): 305–312. DOI: 10.1007/S00344-020-10095-3.

[cit0048] Hayat S., Hayat Q., Alyemeni M.N., Wani A.S., Pichtel J., Ahmad A. (2012) Role of proline under changing environments: A review. Plant Signal. Behav. 7(11): 1456. DOI: 10.4161/Psb.21949. 22951402 PMC3548871

[cit0049] Hogewoning S.W., Wientjes E., Douwstra P., Trouwborst G., Van Ieperen W., Croce R., Harbinson J. (2012) Photosynthetic quantum yield dynamics: From photosystems to leaves. Plant Cell 24(5): 1921–1935. DOI: 10.1105/Tpc.112.097972. 22623496 PMC3442578

[cit0050] Hu F., Zhang Y., Guo J. (2023) Effects of drought stress on photosynthetic physiological characteristics, leaf microstructure, and related gene expression of Yellow Horn. Plant Signal. Behav. 18(1). DOI: 10.1080/15592324.2023.2215025. PMC10228403 37243677

[cit0051] Hura T., Hura K., Ostrowska A., Grzesiak M., Dziurka K. (2013) The cell wall-bound phenolics as a biochemical indicator of soil drought resistance in winter triticale. Plant Soil Environ. 59(5): 189–195. DOI: 10.17221/665/2012-Pse.

[cit0052] Hura T., Hura K., Dziurka K., Ostrowska A., Bączek R., Grzesiak M. (2012) An increase in the content of cell wall-bound phenolics correlates with the productivity of triticale under soil drought. J. Plant Physiol. 169(17): 1728–1736. DOI: 10.1016/J.Jplph.2012.07.012. 22980393

[cit0053] Ijaz S., Haq I.U., Khan I.A., Ali H.M., Kaur S., Razzaq H.A. (2022) Identification of resistance gene analogs of the NBSLRR family through transcriptome probing and in silico prediction of the expressome of Dalbergia sissoo under dieback disease stress. Front. Genet. 13. DOI: 10.3389/Fgene.2022.1036029. PMC9585183 36276980

[cit0054] Islam M.M., Sandhu J., Walia H., Saha R. (2020) Transcriptomic data-driven discovery of global regulatory features of rice seeds developing under heat stress. Comput. Struct. Biotechnol. J. 18: 2556–2567. DOI: 10.1016/J.Csbj.2020.09.022. 33033578 PMC7522763

[cit0055] Javed M., Iqbal M., Bano H., Hussain N., Ghaffar A., Zafar Z.U., Hussain A., Abdullah M., Ayyaz A., Farooq M.A., Ashraf M., Athar H.R. (2021) Photosynthetic acclamatory response of Panicum antidotale Retz. *Populations to root zone desiccation stress.* Braz. J. Biol. 84: e252735. DOI: 10.1590/1519-6984.252735. 34932636

[cit0056] Jung K.H., Han M.J., Lee D.Y., Lee Y.S., Schreiber L., Franke R., Faust A., Yephremov A., Saedler H., Kim Y.W., Hwang I., An G. (2006) Wax-deficient anther1 is involved in cuticle and wax production in rice anther walls and is required for pollen development. Plant Cell 18(11): 3015–3032. DOI: 10.1105/Tpc.106.042044. 17138699 PMC1693940

[cit0057] Kokkinakis D.M., Brooks J.L. (1979) Hydrogen peroxide-mediated oxidation of indole-3-acetic acid by tomato peroxidase and molecular oxygen. Plant Physiol. 64(2): 220. DOI: 10.1104/Pp.64.2.220. 16660936 PMC543058

[cit0058] Landi M., Zivcak M., Sytar O., Brestic M., Allakhverdiev S.I. (2020) Plasticity of photosynthetic processes and the accumulation of secondary metabolites in plants in response to monochromatic light environments: A review. Biochim. Biophys. Acta Bioenerg. 1861(2): 148131. DOI:10.1016/J.Bbabio.2019.148131. 31816291

[cit0059] Le Gall H., Philippe F., Domon J.M., Gillet F., Pelloux J., Rayon C. (2015a) Cell wall metabolism in response to abiotic stress. Plants 4(1): 112–166. DOI: 10.3390/Plants4010112. 27135320 PMC4844334

[cit0060] Le Gall H., Philippe F., Domon J.M., Gillet F., Pelloux J., Rayon C. (2015b) Cell wall metabolism in response to abiotic stress. Plants 4(1): 112–66. DOI: 10.3390/Plants4010112. 27135320 PMC4844334

[cit0061] Li L., Dou N., Zhang H., Wu C. (2021) The versatile GABA in plants. Plant Signal. Behav. 16(3). DOI: 10.1080/15592324.2020.1862565. PMC7889023 33404284

[cit0062] Li Q., Qin X., Qi J., Dou W., Dunand C., Chen S., He Y. (2020) CsPRX25, a class III peroxidase in Citrus sinensis, confers resistance to citrus bacterial canker through the maintenance of ROS homeostasis and cell wall lignification. Hortic. Res. 7(1): 1–11. DOI: 10.1038/S41438-020-00415-9. 33328465 PMC7705758

[cit0063] Lichtenthaler H.K. (1987) Chlorophylls and carotenoids: Pigments of photosynthetic biomembranes. Methods Enzymol. 148(C): 350–382. DOI: 10.1016/0076-6879(87)48036-1.

[cit0064] Liu Y., Han N., Wang S., Chen C., Lu J., Riaz M.W., Si H., Sun G., Ma C. (2021) Genome-wide identification of Triticum aestivum xylanase inhibitor gene family and inhibitory effects of XI-2 subfamily proteins on Fusarium graminearum GH11 xylanase. Front. Plant Sci. 12. DOI: 10.3389/Fpls.2021.665501. PMC8350787 34381472

[cit0065] Liu Z., Bi S., Meng J., Liu T., Li P., Yu C., Peng X. (2022) Arbuscular mycorrhizal fungi enhanced rice proline metabolism under low temperature with nitric oxide involvement. Front. Plant Sci. 13: 962460. DOI: 10.3389/Fpls.2022.962460. 36247649 PMC9555847

[cit0066] Lv P., Zhang C., Xie P., Yang X., El-Sheikh M.A., Hefft D.I., Ahmad P., Zhao T., Bhat J.A. (2022) Genome-wide identification and expression analyses of the chitinase gene family in response to white mold and drought stress in soybean (Glycine max). Life 12(9). DOI: 10.3390/Life12091340. PMC9504482 36143377

[cit0067] Ma L., Kong F., Sun K., Wang T., Guo T. (2021) From classical radiation to modern radiation: Past, present, and future of radiation mutation breeding. Front. Public Health 9: 768071. DOI: 10.3389/Fpubh.2021.768071. 34993169 PMC8725632

[cit0068] Maghuly F., Bado S., Jankowicz-Cieslak J., Laimer M. (2016) Chemical and physical mutagenesis in Jatropha curcas. Biotechnol. Plant Mutat. Breed. 21–38. DOI: 10.1007/978-3-319-45021-6_2.

[cit0069] Mahmood T., Khalid S., Abdullah M., Ahmed Z., Shah M.K.N., Ghafoor A., Du X. (2020) Insights into drought stress signaling in plants and the molecular genetic basis of cotton drought tolerance. Cells 9(1): 105. DOI: 10.3390/Cells9010105. PMC7016789 31906215

[cit0070] Malefo M.B., Mathibela E.O., Crampton B.G., Makgopa M.E. (2020) Investigating the role of Bowman-Birk serine protease inhibitor in Arabidopsis plants under drought stress. Plant Physiol. Biochem. 149: 286–293. DOI: 10.1016/J.Plaphy.2020.02.007. 32097847

[cit0071] Mathur S., Agrawal D., Jajoo A. (2014) Photosynthesis: Response to high temperature stress. J. Photochem. Photobiol. B Biol. 137: 116–126. DOI: 10.1016/J.Jphotobiol.2014.01.010. 24796250

[cit0072] Matsushima R., Maekawa M., Kusano M., Kondo H., Fujita N., Kawagoe Y., Sakamoto W. (2014) Amyloplast-localized substandard starch grain4 protein influences the size of starch grains in rice endosperm. Plant Physiol. 164(2): 623. DOI: 10.1104/Pp.113.229591. 24335509 PMC3912094

[cit0073] Mishra P., Mishra J., Arora N.K. (2021) Plant growth-promoting bacteria for combating salinity stress in plants – Recent developments and prospects: A review. Microbiol. Res. 252: 126861. DOI: 10.1016/J.Micres.2021.126861. 34521049

[cit0074] Mishra S.S., Behera P.K., Kumar V., Lenka S.K., Panda D. (2018) Physiological characterization and allelic diversity of selected drought tolerant traditional rice (Oryza sativa L.) landraces of Koraput, India. Physiol. Mol. Biol. Plants 24(6): 1035–1046. DOI: 10.1007/S12298-018-0606-4. 30425421 PMC6214433

[cit0075] Nakajima S., Ito H., Tanaka R., Tanaka A. (2012) Chlorophyll b reductase plays an essential role in the maturation and storability of Arabidopsis seeds. Plant Physiol. 160(1): 261. DOI: 10.1104/Pp.112.196881. 22751379 PMC3440204

[cit0076] Nakamura T., Ishikawa M., Nakatani H., Oda A. (2008) Characterization of cold-responsive extracellular chitinase in bromegrass cell cultures and its relationship to antifreeze activity. Plant Physiol. 147(1): 391. DOI: 10.1104/Pp.106.081497. 18359848 PMC2330313

[cit0077] Neto A.D.A., Nogueira R.J.M.C., Melo Filho P.A., Santos R.C. (2010) Physiological and biochemical responses of peanut genotypes to water deficit. J. Plant Interact. 5(1): 1–10. DOI: 10.1080/17429140902999243.

[cit0078] Nounjan N., Mahakham W., Siangliw J.L., Toojinda T., Theerakulpisut P. (2020) Chlorophyll retention and high photosynthetic performance contribute to salinity tolerance in rice carrying drought tolerance quantitative trait loci (QTLs). Agric. 10(12): 620. DOI: 10.3390/Agriculture10120620.

[cit0079] Padmavathi T.A.V., Rao D.M. (2013) Differential accumulation of osmolytes in 4 cultivars of peanut (Arachis hypogaea L.) under drought stress. J. Crop Sci. Biotechnol. 16(2): 151–159. DOI: 10.1007/S12892-012-0102-2.

[cit0080] Pfister B., Zeeman S.C. (2016) Formation of starch in plant cells. Cell. Mol. Life Sci. 73(14): 2781. DOI: 10.1007/S00018-016-2250-X. 27166931 PMC4919380

[cit0081] Priatama R.A., Heo J., Kim S.H., Rajendran S., Yoon S., Jeong D.H., Choo Y.K., Bae J.H., Kim C.M., Lee Y.H., Demura T., Lee Y.K., Choi E.Y., Han C.D., Park S.J. (2022) Narrow LPA1 metaxylems enhance drought tolerance and optimize water use for grain filling in dwarf rice. Front. Plant Sci. 13: 894545. DOI: 10.3389/Fpls.2022.894545. 35620680 PMC9127761

[cit0082] Rahim F.H.A., Zainal Abidin N., Hawari N.N. (2017) Analyzing the impact of price subsidy on rice self-sufficiency level in Malaysia: A preliminary finding. AIP Conf. Proc. 1905. DOI: 10.1063/1.5012213.

[cit0083] Rajput V.D., Harish R.K.S., Verma K.K., Sharma L., Quiroz-Figueroa F.R., Meena M., Gour V.S., Minkina T., Sushkova S., Mandzhieva S. (2021) Recent developments in enzymatic antioxidant defence mechanism in plants with special reference to abiotic stress. Biol. 10(4). DOI: 10.3390/Biology10040267. PMC8066271 33810535

[cit0084] Raza A., Razzaq A., Mehmood S.S., Zou X., Zhang X., Lv Y., Xu J. (2019) Impact of climate change on crops adaptation and strategies to tackle its outcome: A review. Plants 8(2): 34. DOI: 10.3390/Plants8020034. 30704089 PMC6409995

[cit0085] Rehman S., Jørgensen B., Aziz E., Batool R., Naseer S., Rasmussen S.K. (2020) Genome-wide identification and comparative analysis of the serpin gene family in Brachypodium and barley. Plants 9(11): 1–19. DOI: 10.3390/Plants9111439. PMC7692276 33114466

[cit0086] Reis A.D.P., Carvalho R.F., Costa I.B., Girio R.J.S., Gualberto R., Spers R.C., Gaion L.A. (2022) Hydrogen peroxide is involved in drought stress long-distance signaling controlling early stomatal closure in tomato plants. Braz. J. Biol. 82: e267343. DOI: 10.1590/1519-6984.267343. 36383799

[cit0087] Rody H.V.S., Bombardelli R.G.H., Creste S., Camargo L.E.A., Van Sluys M.A., Monteiro-Vitorello C.B. (2019) Genome survey of resistance gene analogs in sugarcane: Genomic features and differential expression of the innate immune system from a smut-resistant genotype. BMC Genomics 20(1): 1–17. DOI: 10.1186/S12864-019-6207-Y. 31694536 PMC6836459

[cit0088] Sakamoto T., Kitano H., Fujioka S. (2017) Rice erect leaf 1 acts in an alternative brassinosteroid signaling pathway independent of the receptor kinase OsBRI1. Plant Signal. Behav. 12(12). DOI: 10.1080/15592324.2017.1396404. PMC5792126 29172939

[cit0089] Sekhwal M.K., Li P., Lam I., Wang X., Cloutier S., You F.M. (2015) Disease resistance gene analogs (RGAs) in plants. Int. J. Mol. Sci. 16(8): 19248. DOI: 10.3390/Ijms160819248. 26287177 PMC4581296

[cit0090] Seleiman M.F., Al-Suhaibani N., Ali N., Akmal M., Alotaibi M., Refay Y., Dindaroglu T., Abdul-Wajid H.H., Battaglia M.L. (2021) Drought stress impacts on plants and different approaches to alleviate its adverse effects. Plants 10(2): 1–25. DOI: 10.3390/Plants10020259. PMC7911879 33525688

[cit0091] Shi X., Dong S., Liu W. (2020) Structures of plant resistosome reveal how NLR immune receptors are activated. Abiotech 1(2): 147. DOI: 10.1007/S42994-019-00012-Y. 36304717 PMC9590527

[cit0092] Shreya S., Supriya L., Padmaja G. (2022) Melatonin induces drought tolerance by modulating lipoxygenase expression, redox homeostasis and photosynthetic efficiency in Arachis hypogaea L. Front. Plant Sci. 13: 1069143. DOI: 10.3389/Fpls.2022.1069143. 36544878 PMC9760964

[cit0093] Singh R., Parihar P., Singh S., Mishra R.K., Singh V.P., Prasad S.M. (2017) Reactive oxygen species signaling and stomatal movement: Current updates and future perspectives. Redox Biol. 11: 213–18. DOI: 10.1016/J.Redox.2016.11.006. 28012436 PMC5192041

[cit0094] Tadele Z. (2016) Mutagenesis and TILLING to dissect gene function in plants. Curr. Genomics 17(6): 499. DOI: 10.2174/1389202917666160520104158. 28217006 PMC5282601

[cit0095] Thapa R., Tabien R.E., Johnson C.D., Septiningsih E.M. (2023) Comparative transcriptomic analysis of germinating rice seedlings to individual and combined anaerobic and cold stress. BMC Genomics 24(1): 1–25. DOI: 10.1186/S12864-023-09262-Z. 37024819 PMC10080786

[cit0096] Todkar L., Harikrishna G.P.S., Jain N., Singh P.K., Prabhu K.V. (2020) Introgression of drought tolerance QTLs through marker-assisted backcross breeding in wheat (Triticum aestivum L.). Indian J. Genet. Plant Breed. 80(2): 209–212. DOI: 10.31742/Ijgpb.80.2.12.

[cit0097] Ul Haq S., Khan A., Ali M., Khattak A.M., Gai W.X., Zhang H.X., Wei A.M., Gong Z.H. (2019) Heat shock proteins: Dynamic biomolecules to counter plant biotic and abiotic stresses. Int. J. Mol. Sci. 20(21): 5321. DOI: 10.3390/Ijms20215321. 31731530 PMC6862505

[cit0098] Valera H.G., Mishra A.K., Pede V.O., Yamano T. (2024) Domestic and international impacts of rice export restrictions: The recent case of Indian non-basmati rice. Glob. Food Secur. 41: 100754. 10.1016/j.gfs.2024.100754 PMC11215516 38957381

[cit0099] Vanaja M., Vaidya S. (2015) Variability in drought stress induced responses of groundnut (Arachis hypogaea L.) genotypes. Biochem. Physiol. Open Access 4(1). DOI: 10.4172/2168-9652.1000149.

[cit0100] Wang H., Lu S., Guan X., Jiang Y., Wang B., Hua J., Zou B. (2022) Dehydration-responsive element binding protein 1C, 1E, and 1G promote stress tolerance to chilling, heat, drought, and salt in rice. Front. Plant Sci. 13: 851731. DOI: 10.3389/Fpls.2022.851731. 35685002 PMC9171204

[cit0101] Wang J., Zhang Q., Wang Y., Huang J., Luo N., Wei S., Jin J. (2019) Analyzing the rice young panicle transcriptome reveals the gene regulatory network controlled by Triangular Hull1. Rice 12(1): 1–10. DOI: 10.1186/S12284-019-0265-2. 30725309 PMC6890884

[cit0102] Wang X., Liu H., Yu F., Hu B., Jia Y., Sha H., Zhao H. (2019) Differential activity of the antioxidant defence system and alterations in the accumulation of osmolyte and reactive oxygen species under drought stress and recovery in rice (Oryza sativa L.) tillering. Sci. Rep. 9(1). DOI: 10.1038/S41598-019-44958-X. PMC6561971 31189967

[cit0103] Xie X., He Z., Chen N., Tang Z., Wang Q., Cai Y. (2019) The roles of environmental factors in regulation of oxidative stress in plants. Biomed Res. Int. 2019. DOI: 10.1155/2019/9732325. PMC6530150 31205950

[cit0104] Xiong D., Flexas J. (2018) Leaf economics spectrum in rice: Leaf anatomical, biochemical, and physiological trait trade-offs. J. Exp. Bot. 69(22): 5599–5609. DOI: 10.1093/Jxb/Ery322. 30189099 PMC6255696

[cit0105] Xiong Y., Han Z., Chai J. (2020) Resistosome and inflammasome: Platforms mediating innate immunity. Curr. Opin. Plant Biol. 56: 47–55. DOI: 10.1016/J.Pbi.2020.03.010. 32554225

[cit0106] Yang L., Wang C.C., Guo W.D., Li X.B., Lu M., Yu C.L. (2006) Differential expression of cell wall-related genes in the elongation zone of rice roots under water deficit. Russ. J. Plant Physiol. 53(3): 390–395. DOI: 10.1134/S1021443706030150.

[cit0107] Yang S., Vanderbeld B., Wan J., Huang Y. (2010) Narrowing down the targets: Towards successful genetic engineering of drought-tolerant crops. Mol. Plant 3: 469–490. DOI: 10.1093/Mp/Ssq016. 20507936

[cit0108] Zafar A., Hameed A., Khan A.S., Ashraf M. (2017) Heat shock induced morpho-physiological response in indica rice (Oryza sativa L.) at the early seedling stage. Pak. J. Bot. 49(2): 453–463.

[cit0109] Zhu X., Xiong L. (2013) Putative megaenzyme DWA1 plays essential roles in drought resistance by regulating stress-induced wax deposition in rice. Proc. Natl. Acad. Sci. U.S.A. 110(44): 17790–17795. DOI: 10.1073/Pnas.1316412110. 24127586 PMC3816433

